# Supplementary material for: Structural Analysis and Whole Genome Mapping of a New Type of Plant Virus Subviral RNA: Umbravirus-Like Associated RNAs
Source: Viruses. 2021 Apr 9;13(4):646. doi: 10.3390/v13040646 (PMC8068935; doi:10.3390/v13040646)
Supplement: Supplementary file 1 [file viruses-13-00646-s001.pdf]

```
Consensus MP motif   xxxLGxxxxxxxxxxxxxxxxxxxxUxxNxxxxxxxxxxxxxxPxxxxxxxUxxxxxxxx
EMaV-2 (53-109)      SAQLNQEAVAYSIPCNVRNMPTITTLATNPLFWRITAITVAMEPAKSTSTQIAGV---GN
EMaV-1 (53-109)      SAQLGQEAVAYSIPCNVRNMPTILTLATTPLYWRITAITVAMEPAKSTSTQIAGV---GN
OULV   (53-109)      STTTGKEAVWAIWPINVGVFPQLVTAVSDPLYWRVTSVTIAMEPAMSTSTQHCGV---GL
SULV   (58-114)      SVQLGQEAVAYTVPLNLTGIPSIKDLATNPLYYRITSVMIAMEPALSTSTQICGV---GN
SbaVa  (51-95)       HKWV--TELVSSAPYKGGRFKTLSTTVD------------VVGPTAK-AEIYVGIIRTPP

Consensus MP motif   xxxxxxxxxxxxxxxxxxxxxxxxxxxxxxG&xUxVxxxUxxx&xxxxxxxxxxxxUxxL
EMaV-2 (110-169)     ADSYQSATFNGFGNVFKKLRALNYVRRSAPGGNLQVRWPINMDWISASSAASDSTQVPSL
EMaV-1 (110-169)     ADSFQSATYNGFGNVFKKMRALNFVRRSAPGGNLQVRWPINMDWISASDTDKDSTKVPSL
OULV   (110-169)     ATSGAYSSGEGFGNLFKYKRACNYTRRSPVGGNVMVKWPISMPYSLNDDAHKSTGLTAGI
SULV   (115-172)     ADYYTTRTYNGFGNVFKKMRTLHFSKRSTPGGNVQVKWPINMEWVKITD--TNTLTVPQL
SbaVa  (96-152)      DTVKPMGKENDWSLIIRLWRDCYIRRKSTGSSGLACNFNLTTPWMTDDS---NEDQTASV

Consensus MP motif   xxxxxxxxxxxxxxxxxxxxxx&xxxUxxxxG
EMaV-2 (170-202)     FFAVTNPGVIETKQGDSEAWLEWELELEFIVGG---------------------------
EMaV-1 (170-202)     FFAVTNPGVIETKQGDSEAWLEWELELEYIVGG---------------------------
OULV   (170-197)     VIGVTNPGAIT-----GLAWVEISLNVEYVMGT---------------------------
SULV   (173-205)     FFAVTNPGVIETKAGEHESWFEFELAIQYIIGG---------------------------
SbaVa  (153-207)     FIGWYNDGLTNSRAS-----VDMRVRVACIPNRLWPEGTTGKGHMRNKRGLEEVDMKTHE
```

Supplementary Figure S1. Alignment of ORF5 amino acid sequences. Alignment was built using Clustal Omega in Uniprot (https://www.uniprot.org/). Consensus movement protein (MP) residues as reported in Mushegian and Koonin, 1993 are indicated. In the consensus sequence, aliphatic and hydrophobic (aliphatic or aromatic) aa are designated by *U* and *&*, respectively, and non-consensus aa are designated by *x*. Conserved residues and motif found in 30K MP superfamily are in bold. Orange and purple denote consensus aliphatic and hydrophobic aa. EMaV-1: Ethiopia maize-associated virus (MN715238); EMaV-2: Ethiopia maize-associated virus (MF415880); OULV: opuntia umbra-like virus (MH579715); SULV: sugarcane umbra-like virus (MN868593); SbaVA: strawberry associated Virus A (MK211274).

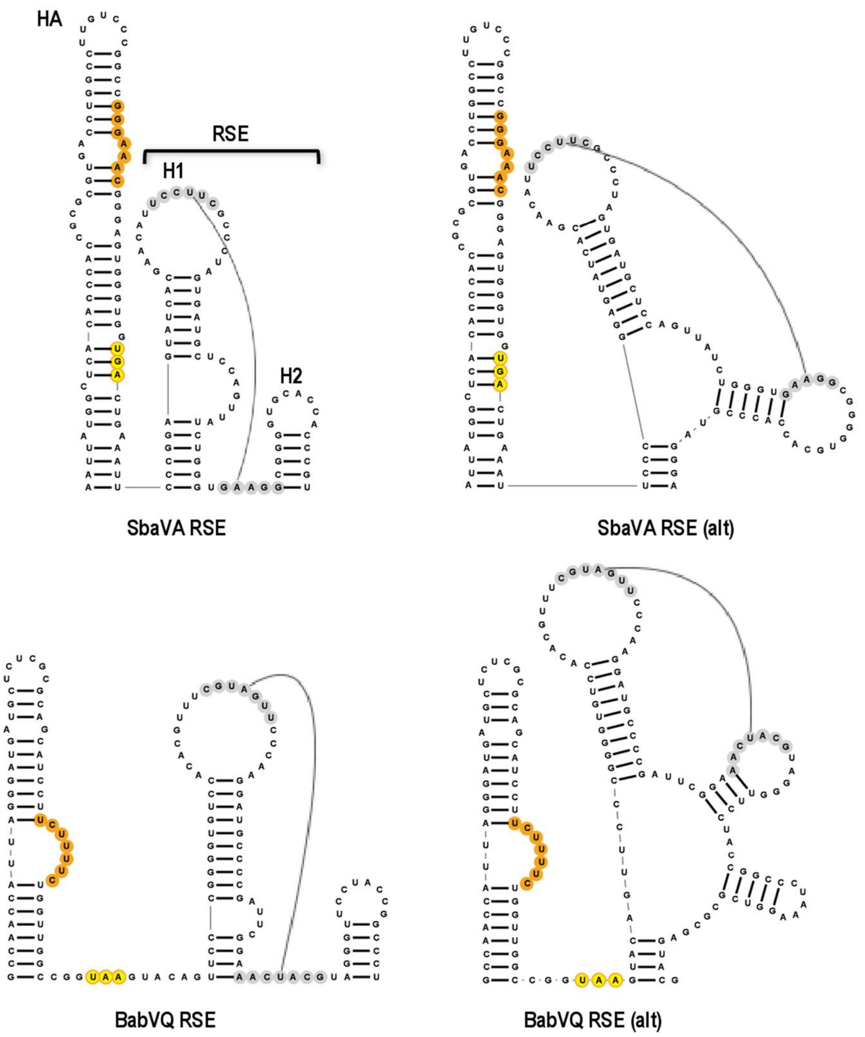

Supplementary Figure S2. Predicted structures at the -1PRF site for Class 3 SbaVA and Class 1 BabVQ. Both putative conformations are shown. Slippery site is in orange and stop codon is in yellow. Structurally conserved local pseudoknot is shown, which would negate the ability of the H1 residues to participate in a long-distance interaction with complementary sequence at the 3' end.

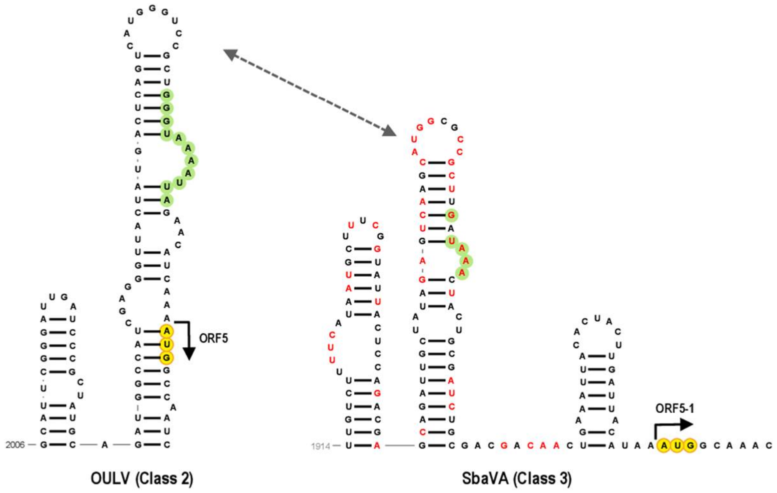

Supplemental Figure S3. Similar structures upstream of Class 2 ORF5 and Class 3 ORF5-1. Residues in red in SbaVA denote sequence conservation with OULV. CCS sequence normally found at the 5' end of umbravirus sgRNAs is shaded green. Translation initiation site for ORF 5 and ORF5-1 are shaded yellow.

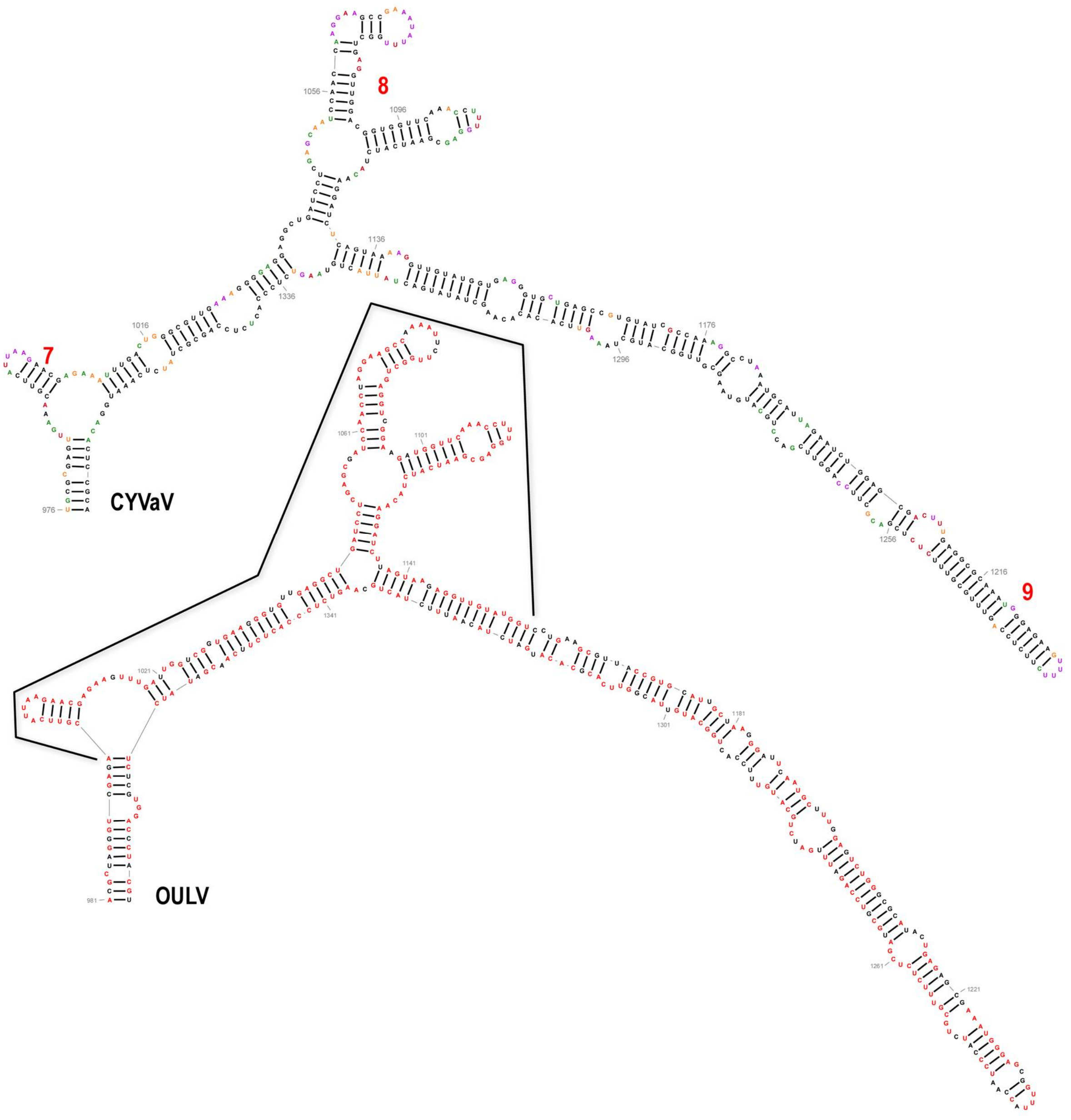

Supplementary Figure S4. Strongly conserved sequence/structure in D2 of CYVaV and OULV. SHAPE data is shown on the CYVaV structure. Red bases in OULV are identical to those of CYVaV. Note the exceptionally strong conservation encompassing structures 7 and 8 (line). The function of these structures is not known.

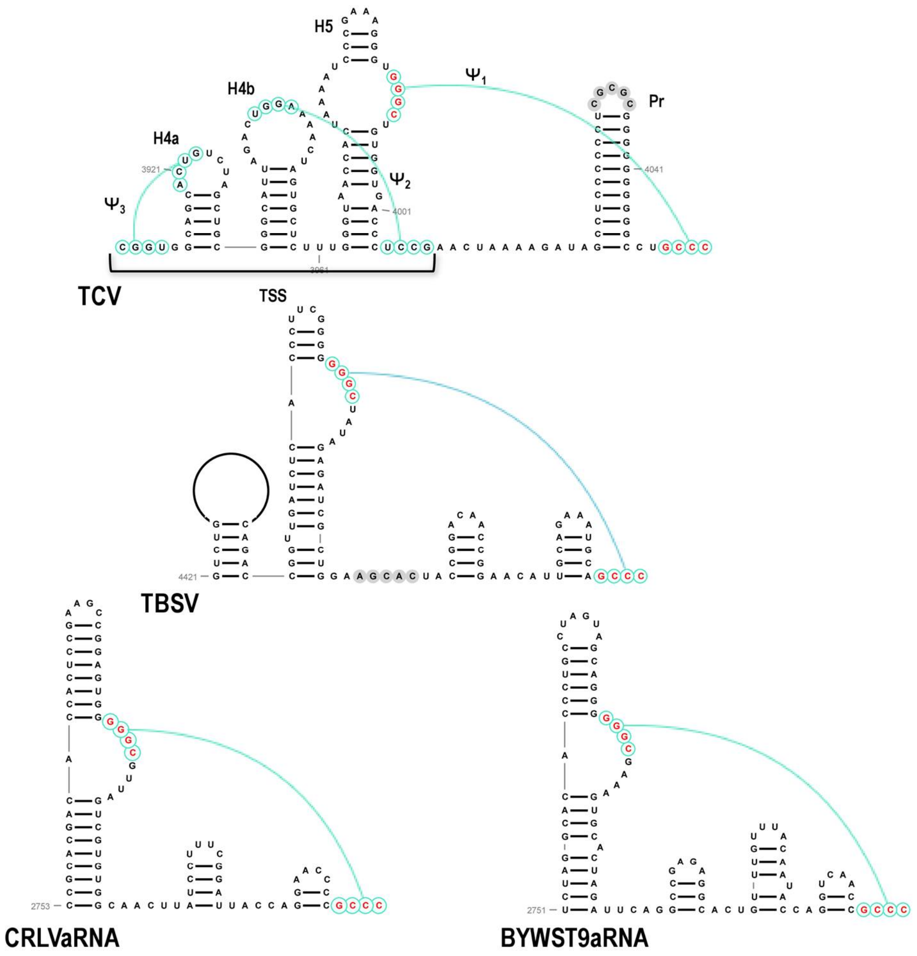

Supplementary Figure S5. 3′ terminal region of TCV, TBSV and two tlaRNAs. Note that the 3′ end of the betacarmovirus TCV is similar to that of many umbraviruses and Class 2 and Class 3 ulaRNAs (Fig. 13) but with a distinctively different H5. The 3′ ends of tombusviruses like TBSV and all tlaRNAs are also structurally similar, supporting their evolutionary relationship as shown in Fig. 2. Residues shaded in gray connect in an RNA: RNA interaction with the RSE element to promote ribosomal read-through. Corresponding residues for the tlaRNAs have not been examined.
